# Dataset from de novo transcriptome assembly of *Nephelium lappaceum* aril

**DOI:** 10.1016/j.dib.2018.12.034

**Published:** 2018-12-14

**Authors:** Deden Derajat Matra, Arya Widura Ritonga, Azis Natawijaya, Roedhy Poerwanto, Sobir, Winarso Drajad Widodo, Eiichi Inoue

**Affiliations:** a Department of Agronomy and Horticulture, Bogor Agricultural University, Indonesia; b Research and Development Division, Mekarsari Fruit Garden, Indonesia; c College of Agriculture, Ibaraki University, Japan

## Abstract

*Nephelium lappaceum* (Rambutan), is one of tropical fruit in which - cultivated widely in Indonesia and has good taste and aroma. However, the transcriptomic study of rambutan has limited. In this study, we performed transcriptome assembly using paired-end Illumina technology. The assembled transcriptome was constructed using Trinity and after filtering and removal sequences redundancy produced 36,303 contigs. The contig ranged 201–11,770 bp and N50 has 1327 bp. The contig was annotated with several databases such as SwissProt, TrEMBL, and nr/nt of NCBI databases. The raw reads are deposited in the DDBJ with DRA accession number, DRA007359: https://www.ncbi.nlm.nih.gov/sra/?term=DRA007359. The assembled contigs of transcriptome are deposited in the DDBJ TSA repository with accession number IADQ01000001–IADQ01036303: ftp://ftp.ddbj.nig.ac.jp/ddbj_database/tsa/IADQ.gz and also can be accessed at http://rujakbase.id.

**Specifications table**

**Table:** 

Subject area	Agricultural and Biological Sciences
More specific subject area	Horticulture
Type of data	RNA sequencing data
How data was acquired	Illumina HiSeq X Ten
Data format	Raw sequencing reads and assembled contigs
Experimental factors	RNA sequencing was performed by using Illumina HiSeq X Ten
Experimental features	RNA sequencing of aril tissue at ripening stage
Data source location	Cileungsi, Bogor, West Java, Indonesia (6°24′50.1′′S 106°59׳05.7′′E)
Data accessibility	The raw data have been deposited in the DNA Data Bank of Japan (DDBJ) under the DRA accession number, DRA007359 and the assembled contigs of transcriptome have been deposited in the DDBJ TSA repository with accession number, IADQ01000001-IADQ01036303 and also can be accessed at http://rujakbase.id
Related research article	Lim T.K., *Nephelium lappaceum*. In: Edible Medicinal and Non-Medicinal Plants, Springer, Dordrecht, 2013

**Value of the data**

• This data provides transcriptome for the first time of *Nephelium Lappaceum* from aril fruits.
• This data will be useful to obtain molecular markers such as microsatellite and single nucleotide polymorphisms for breeding and selection of new cultivars in *Nephelium Lappaceum* and related-genus.
• This data will further be valuable for gene expression analysis using treatments among their species and related-genus.

## 1 Data

*Nephelium lappaceum* L. (rambutan) is originated from Indonesia and Malay Peninsula [1]. In this study, a de novo transcriptome assembly of *Nephelium lappaceum* has been reported. The transcriptome data were obtained from the aril part of the fruit. The aril tissue was collected, and the high quality of RNA was extracted for paired-end sequencing technology of Illumina. The high quality of reads was obtained, and de novo assembly was performed using Trinity v.2.4.0 [2]. All statistics of reads and assembled sequence were determined (Table 1). The contigs were reconstructed using CAP3 [3] and CD-HIT-EST v.4.6.8 [4] to remove redundant contigs and then the contigs were filtering and clustering using Corset v.1.06 [5]. The contigs were annotated with several databases using the BLAST+ v.2.7.1 program [6]. An overview of the sequencing assembly of *Nephelium lappaceum* transcriptome data is presented in Table 2.

**Table 1:** Read and assembly statistics of Rambutan (*Nephelium lappaceum* L.) aril.

Features	Numbers
Reads and bases (bp)	60,133,100 / 9,019,965,000
Number and bases total (bp) of transcripts	113,476 / 81,896,149
Number and bases total (bp) of unigenes	65,028 / 38,541,702
Number and bases total (bp) of contigs	36,303 / 39,058,626
Length range, average, and N50 of transcripts (bp)	201–11,770 / 721.70 / 1075
Length range, average, and N50 of unigenes (bp)	201–11,770 / 592.69 / 854
Length range, average, and N50 of contigs (bp)	201–11,770 / 1075.91 / 1327

**Table 2:** Functional annotation of rambutan (*Nephelium lappaceum* L.) contigs.

Database source	Number of contig (percentage)
Contig Number	36,303
– Non-redundant protein (nr) NCBI	29,619 (81.58%)
– Non-redundant nucleotide (nt) NCBI	25,453 (70.11%)
– SwissProt UniProt	21,563 (59.39%)
– TrEMBL UniProt	30,232 (83.28%)

## 2 Experimental design, materials, and methods

Rambutan var. Binjai were collected from Mekarsari Fruit Garden at ripening stage. The flesh aril was used for RNA extraction. The total RNA was extracted using ISOLATE RNA (Bioline) following the protocol. The quality and quantity of DNA were checked by P360 Nanophotometer (Implen, München, Germany). The extracted RNA was subjected to preparation of a paired-end library for RNA sequencing using the Illumina Hiseq X Ten (BGI, Hongkong). After sequencing, the raw reads were filtered. Data filtering includes removing adaptor sequences, contamination and low-quality read from raw reads. The high quality of reads used to construct assembled transcriptome using Trinity package with default parameters and minimum length of 200 bp. The assembled contigs were performed by CAP3 (−p 90), and CD-HIT-EST (−c 0.90 −M 0 −T 0) and clustering with Corset after filtering low expression reads below 1 CPM. Several databases such as nt and nr databases from NCBI and SwissProt and TrEMBL databases from UniProt were used to annotate the contigs using the BLAST+ program with the cut-off of 10^−5^ [7].

## 3 Data accessibility

All raw data and sequences have been deposited to the DDBJ with accession number DRA007359: https://www.ncbi.nlm.nih.gov/sra/?term=DRA007359 and assembled contigs have been deposited to the Transcriptome Shotgun Assembly (TSA) with accession number, IADQ01000001–IADQ01036303: ftp://ftp.ddbj.nig.ac.jp/ddbj_database/tsa/IADQ.gz and also can be downloaded at http://rujakbase.id/content/download.
